# Health Planning in Times of COVID-19 in Burkina Faso: The Role of Its National Strategic Pandemic Management Committee

**DOI:** 10.1177/2752535X241256414

**Published:** 2024-05-30

**Authors:** Thomas Druetz, Frank Bicaba, Cissé Zainabou, Abel Bicaba

**Affiliations:** 1 Department of Social and Preventive Medicine, School of Public Health, 5622 University of Montreal, Montreal, QC, Canada; 2 Centre de Recherche en Santé Publique, Montreal, QC, Canada; 3 Société d’Études et de Recherches en Santé Publique, Ouagadougou, Burkina Faso; 4 University Aix-Marseille, Centre d’Etudes et de Recherche sur les Services de Santé et la Qualité de Vie, Marseille, France

**Keywords:** COVID-19, health policy, Burkina Faso, crisis management, epidemic, strategic planning

## Abstract

**Context:**

Presenting the COVID-19 crisis as a pandemic misleadingly implies a certain homogeneity between the regions of the Globe in terms of their burden and reactions. However, from the outset of the crisis, countries presented different epidemiological realities and sometimes adopted divergent, even opposing measures. Curiously, the heterogeneity of responses persisted as scientific evidence accumulated about COVID-19 and the strategies for dealing with it.

**Case study:**

This commentary aims to recount the specific experience of Burkina Faso, and how it reoriented its initial biomedical response into a multisectoral strategy. Burkina Faso set up a committee specifically to examine the effects not only of the pandemic, but also of the control measures. This committee was mandated to decompartmentalize the lens through which the COVID-19 was dealt with. It entered into dialogue with a level of stakeholders often overlooked during national health crisis: communities. As a member of this “National Committee for Crisis Management of the Pandemic”, one of the co-authors contributed to its orientations and has witnessed first-hand some of the challenges it faced.

**Recommendations:**

This experience suggests that the project of extricating the field of public health from medicine is advancing in Burkina Faso. In order to manage future crises more effectively and across different sectors, there is an urgent need to establish state structures and to strengthen public health systems. States need coordination units that have the legitimacy, authority and resources required to mobilize a variety of actors at the community, national and international levels.

## Teaser

In Burkina Faso, the national strategic multisectoral committee set up to manage the COVID-19 pandemic laid the foundations of a new paradigm: a “new public health” that is no longer at the service of (or governed by) medicine but is resolutely interdisciplinary and built in partnership with communities.

## Key Messages

• Given the complex nature of the pandemic, a multisectoral committee was established in Burkina Faso to mobilize the country’s stakeholders to rapidly counter the spread of the epidemic while limiting its socioeconomic consequences.
• The governmental response in Burkina Faso stands out in two respects: on the one hand, in terms of the influence of community dynamics and, on the other, in terms of an economic recovery plan that took into consideration the significant socio-economic inequities and the risk of their deterioration.
• The pandemic illustrated that in order to manage future crises effectively and across sectors, Burkina Faso needs a public coordination unit that acts as an interface and has the authority, legitimacy, resources and established mechanisms to rapidly mobilize a variety of structures and actors at different levels of decision-making and implementation.

## Background

Since December 2019, the SARS-CoV-2 virus has been detected on all continents and has resulted in the confirmed death of roughly 7 million people according to the World Health Organization (WHO). The official mortality burden has been considerably lower in Africa than in the Americas, Europe, or Asia.^1,2^ Several hypotheses have been put forward to explain this phenomenon, including climatic conditions (heat, humidity), the younger demographic composition of the continent, prior exposure to coronavirus infections, the effectiveness of epidemic control measures, the strengthened immune system of the inhabitants, and their lifestyle (daily time spent outdoors, mobility patterns, etc.).^3,4^ However, the apparently low burden is itself questionable: Not only was the availability of diagnostic tests very limited in many African countries, but treatment-seeking practices in the population, state registration of confirmed cases, and official reports of deaths differed greatly between some African countries and other nations across the world, suggesting that the true burden of COVID-19 is underestimated in sub-Saharan Africa.^5^

Burkina Faso is a landlocked country located in West Africa whose name means “Country of Honest Men”. A true Sahelian crossroads, it borders six other countries in the region: Niger, Mali, Côte d'Ivoire, Benin, Togo and Ghana. According to the most recent World Bank data, Burkina Faso’s population is approximately 21 million and the agricultural sector employs >80% of the total workforce. With a land area of 274,000 km^2^, Burkina Faso ranks 182^nd^ out of 187 on the Human Development Index. Its health system is pyramidal, with 1760 primary health centers, 47 medical centers with surgical facilities, and 13 referral hospitals. Annual government spending on health in 2018 was US$330 million, roughly 10% of the total government budget.

COVID-19 seems to have had major macroeconomic repercussions, as suggested by the 7% decline in economic growth (5.6% in 2019 vs −2.0% in 2020). On April 2, 2020, the government adopted a US$291 million pandemic response plan, of which US$53 million came from the regular state budget.

Box 1: Main characteristics of Burkina Faso and its Health System.

In Burkina Faso (see box and Map), a few centres focused on respiratory infections, notably a surveillance system for severe acute respiratory infections set up in 2016 and two advanced surveillance sites for influenza syndromes. There was also a National Reference Laboratory for Influenza capable of analyzing nasopharyngeal or oropharyngeal swab samples.^6^ It was this laboratory that first identified COVID-19 in Burkina Faso on March 9, 2020. Since this first infection detected, transmission levels have varied over time, marked by a succession of “waves” as observed in many other countries.^7^

**Map 1.**
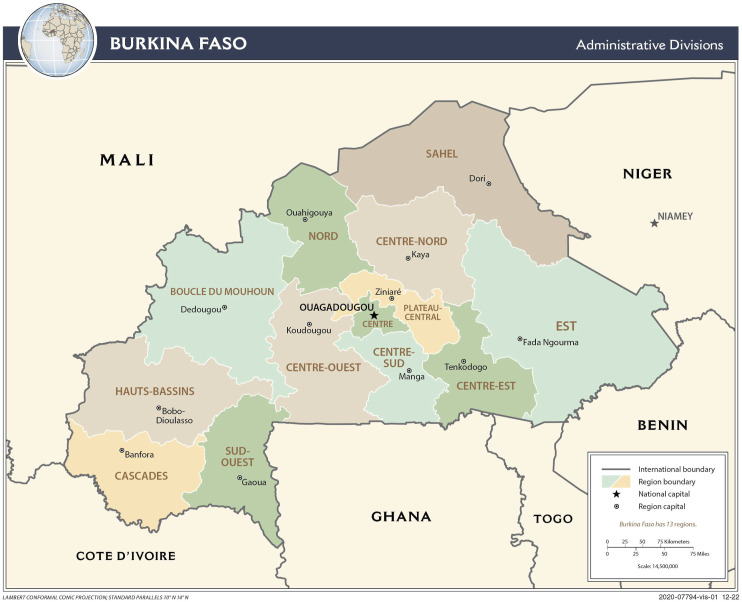
Map of Burkina Faso and its regions.

The Burkina Faso Ministry of Health, in collaboration with other ministerial sectors and technical and financial partners, developed in March 2020 an epidemic preparedness and response plan. This plan was inspired by the one drawn up in 2014 to prepare for the possibility of an extension to Burkina Faso of the Ebola epidemic then underway in four West African countries. These plans mainly consisted in assessing risk and identifying resources, priority problems and, finally, potential activities to be implemented with their budgetary implications. The initial COVID-19 plan was quickly revised (in April 2020) based on Ministry of Health models suggesting that large-scale population spread was likely unless more vigorous interventions were implemented.

Both the plan for the hypothetical management of the Ebola epidemic and the plan for the management of COVID-19 provided for a decision-making structure established by an inter-ministerial decree. In practice, however, this structure had not yet been tested. The aim of this commentary is to retrace the evolution of the pandemic situation in Burkina Faso, and the adaptation of the mandate and modus operandi of its crisis management structure.

## The COVID-19 Crisis in Burkina Faso

### First Outbreak

First, Burkina Faso was more severely affected by COVID-19 than other countries in the region. During the first wave in March-May 2020, the country had the highest mortality rate in West Africa.^7,8^ Based on previous experiences with infectious disease epidemics, the authorities anticipated a rapid and uncontrollable acceleration in transmission as soon as the first case was notified.

While Burkina Faso has certain environmental and demographic characteristics that could slow the spread of COVID-19 and limit mortality rates, it also has a high prevalence of immunosuppressed people (largely due to previous malnutrition, AIDS and tuberculosis epidemics) and fragile health systems.^9,10^ Given the difficulties of ensuring adequate management of severe cases in intensive care, case fatality was predicted to be high.^11^ Authorities prioritized increasing testing rates by strengthening diagnostic capacity and working to ensure isolation and follow-up for confirmed cases.

### Evolution of the Pandemic

**Figure 2.**
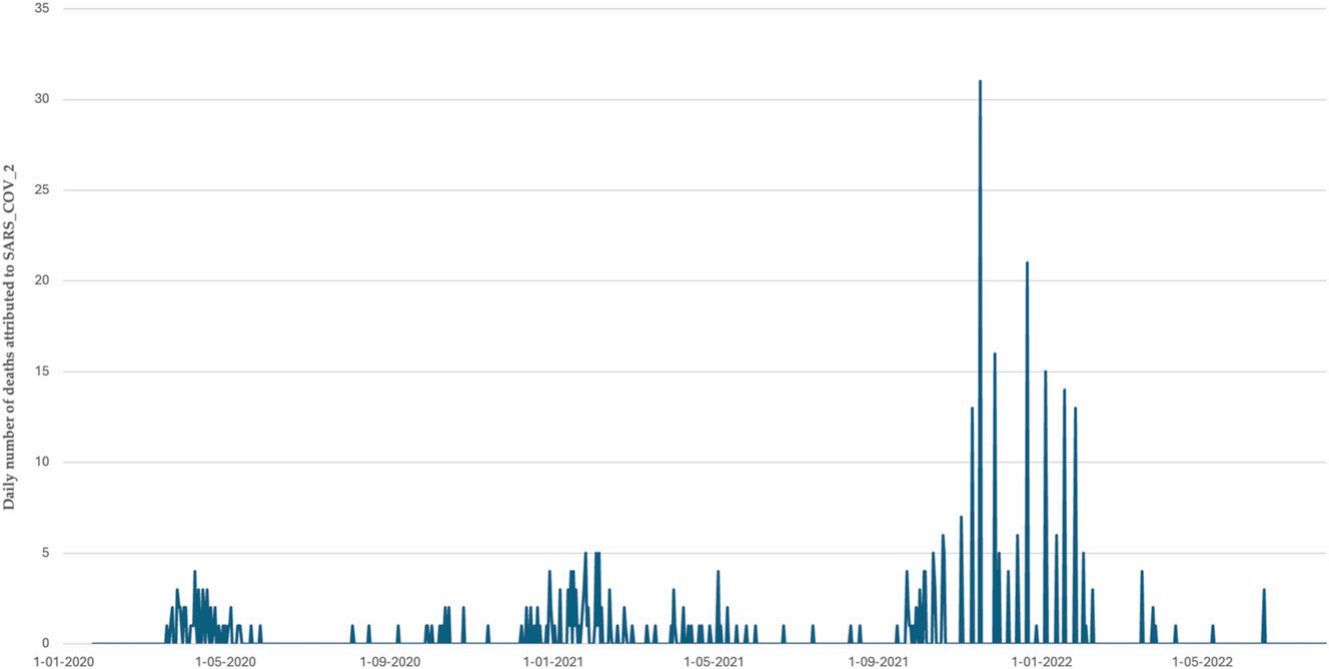
Trends of deaths attributed to COVID-19 in Burkina Faso.

**Figure A1.**
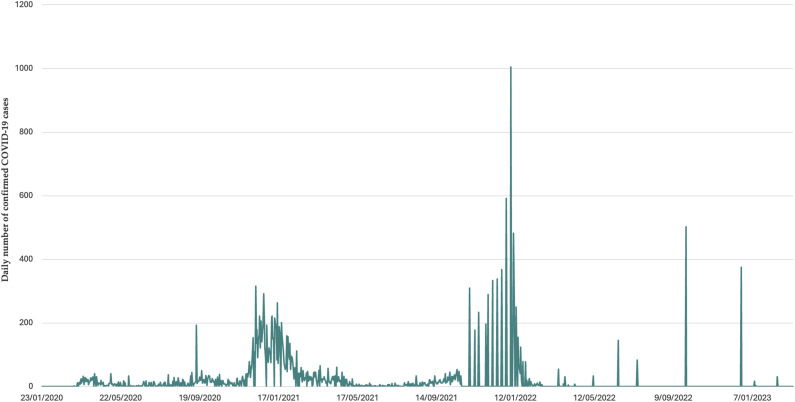
Trends in daily confirmed cases of COVID-19 in Burkina Faso.

There have been three waves of the pandemic in Burkina Faso (see Figure 2). The first wave (March–May 2020) was characterized by a rapid increase in transmission following the detection of the first cases. It was short-lived: The number of cases fell rapidly, and there were only five deaths per week by May 2020. This was followed by a lull marked by an overall decrease in the number of new cases, a steady growth in the number of recoveries, and the stabilization of the number of deaths. From May to December 2020, Burkina Faso recorded ∼150 cases per week and <5 deaths per week (0.7 cases per 100,000 inhabitants and 0.02 deaths per 100,000 inhabitants) (Appendix Figure A1).

The second wave peaked in January 2021, with more than 1000 cases and up to 15 deaths per week. This wave took longer than the first to return to a plateau of low transmission, and May–September 2021 saw cases become sporadic again, with an average of one death per month. Vaccination against COVID-19 has been introduced during this phase, starting in June 2021. However, 3 months after deployment, only 0.02% of the population was reportedly fully vaccinated.^12^ Even after 9 months, vaccination coverage barely reached 5%.^13^ In October 2023, it was estimated that 22% of the population received at least one dose of the vaccine against COVID-19 (source: https://coronavirus.jhu.edu/region/burkina-faso).

The third wave was the most intense in terms of both case and death numbers. In 6 months (September 2021–February 2022), Burkina Faso more than doubled the total number of COVID-19-related deaths recorded since the start of the pandemic. After this peak, transmission returned to low levels (1–2 deaths per month), and there has been no resurgence in morbidity or mortality since March 2022.

According to data from the Pandemic Situation Report of the “Centre des Opérations de Réponse aux Urgences Sanitaires” (https://www.corus.gov.bf/contact), 9 of Burkina Faso’s 13 regions have had at least one positive case since the beginning of the pandemic. The Centre region (home to the capital Ouagadougou) accounts for ∼85% of the cumulative number of cases detected throughout the country, followed by the Hauts Bassins region (home to Bobo-Dioulasso, the country’s second-largest city) with ∼10%. The Centre-West, the East, the Centre-East and the North regions have not recorded any cases. Only 35% of the health districts (25/70) recorded at least one death.

### An Undifferentiated Initial Response Reveals Systemic Shortcomings

From the onset of the epidemic in March 2020, Burkina Faso gradually implemented a series of population-based measures to limit the spread of the virus: a national curfew, border closures, mandatory masking for all, restriction of urban and interurban traffic, quarantines in certain cities, prohibition of gatherings of people, and closure of public markets, schools, educational institutions, and places of worship.^14^ These measures lasted 1-3 months. Authorities also temporarily suspended several health activities and programs that required the mobilization of teams or gathering of community members; in fact, as recommended by the WHO, mass vaccination campaigns for children were suspended, although they quickly resumed.^15–17^ Informational campaigns to educate citizens on COVID-19 were launched in communities and on television and radio.

This response plan reflects the fragility of the health system in Burkina Faso, particularly in terms of preparation for and control of respiratory epidemics. One of the first priorities was to increase the number of health facilities with the material and human resources necessary to perform polymerase chain reaction (PCR) tests; however, laboratory resources were so limited that diagnostic capabilities were deeply affected. At the same time, an insufficient number of hospital beds, a shortage of qualified staff and a lack of equipment compromised the therapeutic management of cases; At the beginning of the pandemic, there were only 11 ventilators in the entire country.^18^ Given these severe limitations to medical care, response to the pandemic was largely focused on preventive public health measures. There was no coordinated reallocations of health personnel or in-depth reorganization of services, as these were of limited use in the management of the disease.

In terms of governance, management, coordination, and communication by health authorities also proved to be flawed. Policies at different levels of government were often contradictory, incoherent, and fragmented. The roles and responsibilities assigned to the stakeholders involved in managing the pandemic were unclear and imprecise, leading to conflicts of authority and leadership and resulting in an inefficient strategic response both on paper and in practice. This situation was later improved with the creation of a multisectoral strategic committee (discussed in detail below).

This vulnerability of the system was quickly compounded by supply difficulties, including shortages of materials needed to carry out diagnostic tests, personal protective equipment (PPE), and supplies needed for therapeutic management. At the global level, there was fierce competition to obtain certain goods produced abroad: The most powerful governments were able to ensure a continuous supply for their citizens, to the detriment of lower-income countries.^19–21^ Unequal access to gloves, masks, swabs, oxygen, and later vaccines and rapid diagnostic tests reflected and exacerbated existing global health inequities.^22^

Obstacles like the closure of borders, slowdown in global air travel, and logistical difficulties in importing goods were all particularly disadvantageous for sub-Saharan African countries, which are less connected in international trade flows.^23^ This trade isolation was particularly damaging to landlocked countries with no port infrastructure, such as Burkina Faso.^24^

At the national level, Burkina Faso’s limited industrial production system provided additional obstacles. While consumption of goods increased during the pandemic and imports slowed in the short to medium term, industrial production could not be increased or redirected as quickly as elsewhere. Import dependence played and continues to play an amplifying role in this supply crisis. Medical and pharmaceutical products have been particularly affected; sampling equipment, PPE and other medical protection, surgical gloves and masks, reagents, laboratory equipment, and other crucial supplies were and are especially difficult to obtain. Pharmaceuticals considered low priority in high-income countries fell victim to shortages when global production was reduced to free up resources; for example, reallocation of facilities and supplies led to shortages of yellow fever vaccine, which constitutes a severe public health threat in low-income countries.^25–27^ Although not yet formally documented in Burkina Faso, shortages of other goods crucial to public health have been reported in several countries in the region, including contraceptive methods, agricultural supplies, and food products.^14,28,29^

## Reorienting the Response

### A Pandemic with Multisectoral Repercussions

The direct impact of the pandemic on health in Burkina Faso has been minimal to date in terms of the number of infections (∼22,000 cases and ∼400 deaths), but indirect and lasting effects cannot be overlooked. Restrictive measures taken to limit transmission, including disruption of vaccination and door-to-door outreach campaigns, may have contributed to the resurgence of other infectious disease outbreaks.^30^ The closure of schools, markets and public places, the curfews, and the quarantine of some towns negatively impacted household income, domestic transportation, food supply, and trade – with potential repercussion on malnutrition.^31^ Although these measures were transient and did not last more than 1-3 months, the climate of uncertainty and fear has increased stress at the individual level. This arguably affected indicators as diverse as the desire to have children, reproductive health practices, domestic violence, academic success, employment decisions, and other aspects that reach well beyond health.^14,32^

In response, authorities concentrated their efforts on developing a plan that acts on all external stressors generated by the epidemic. Economic slowdown, household poverty, and the shutdown of certain industries (particularly tourism) were of particular concern to decision-makers as they confronted the ongoing indirect effects of the emergency measures taken in early 2020 to combat the pandemic.

### Establishment of the National Committee

Given the complex nature of the crisis, a multisectoral committee was established in May 2020 to mobilize the country’s stakeholders to rapidly counter the spread of the epidemic while limiting its socioeconomic consequences. The National Committee for Crisis Management of the Pandemic (NCCMP) was chaired by the Prime Minister and composed of ministries that had a leading role in both the fight against the pandemic and the mitigation of its effects on the population. It also included representatives from technical and financial partners, diplomatic corps, and civil society organizations. Its mandate was to strategically coordinate the management of the crisis and its consequences at the public health and socioeconomic levels.

The NCCMP was responsible for (i) coordinating all actions within the framework of the management of the crisis related to the pandemic; (ii) providing the sectoral orientations necessary for the efficient management of the different parameters of the crisis; (iii) anticipating future risks and threats, studying their consequences on the life of the nation and proposing solutions to the government; (iv) approving sectoral and regional response plans; (v) monitoring and evaluating the implementation and effects of sectoral plans. The NCCMP had an Executive Secretariat composed of six members; this structure served as its operational “arm” and was headed by the Secretary General of National Defense.

This NCCMP was itself made up of 5 sectorial committees: health, civil liberties and community response, cooperation and development, humanitarian assistance, and communication. Although each of these committees had specific areas of intervention, they were in constant interaction and placed directly under the coordination of the NCCMP. Similarly, there were also different regional committees to ensure that information exchanges to and from the central level remain fluid across the country.

## The National Pandemic Crisis Response Plan

From the beginning of its mandate, the NCCMP worked to develop a comprehensive pandemic response plan of unprecedented magnitude. Previous instruments (such as the “Plan d’organisation des secours” or the different outbreak response plans specific to measles, meningitis, etc.) were mainly concerned with the management of limited outbreaks or epidemics, largely focused on therapeutic management protocols within a biomedical perspective. At the time of the COVID-19 crisis, the NCCMP’s objective was to draw up a plan containing new guidelines for managing not an epidemic, but a *pandemic* with multisectoral ramifications. This new *modus operandi* required a reference document that integrated the various aspects (health, social, economic, and psychological, among others) of the crisis.

**Figure 3.**
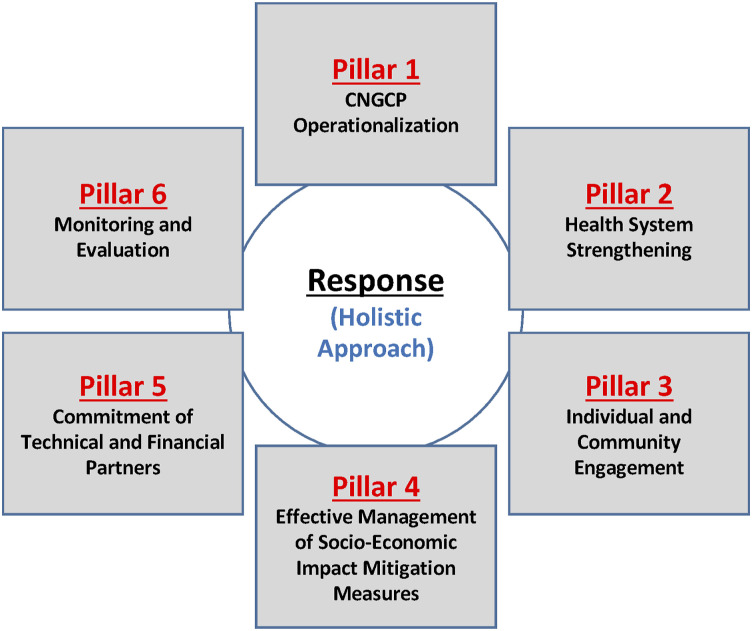
Reference model of the national Covid-19 pandemic crisis response plan.

The National Pandemic Crisis Response Plan was the result of this collaborative process. It is a guiding instrument whose aim is to define and implement a comprehensive, relevant, and cohesive national response to a multifaceted public health crisis. The plan has six pillars: I) the operational component (functioning of the NCCMP); II) health system strengthening; III) individual and community engagement; IV) the interventional component (i.e., the effective management of mitigation measures); V) commitment of technical and financial partners; and VI) monitoring and evaluation (Figure 3).

In addition to the response plan, a roadmap was developed to guide the daily actions of all participants. To enhance the effectiveness of the multisectoral response, five sectoral committees were set up to bring together the various actors from the ministries, the civil society, the main religious communities and customary authorities. These sectoral actions were coordinated by the NCCMP’s Executive Secretariat.

The committee’s interventions have, for example, given rise to a campaign entitled “Remobilizing the whole Nation to step up the fight against Covid-19”. This inclusive campaign helped to involve local customary authorities and communities more closely in the fight against misinformation about COVID19. Government support for radio messages was also provided, and health authorities at different levels were mobilized to support the campaign.

### Implementation Difficulties

Challenges plagued the plan’s implementation at all levels. Within Pillar III (“Individual and Community Engagement”), the NCCMP encountered resistance to compliance with the recommended preventive measures, and enforcement was often inconsistent or inappropriate. For example, recommendations to wear masks were almost systematically ignored by the population, even when supplies (e.g., free masks) were provided for this purpose. The physical distancing of individuals in public places has also been little respected, as well as the use of soap at the entrance to public buildings or places of assembly.

Pillar I (“NCCMP Operationalization”) also encountered several obstacles. While the NCCMP, chaired by the Prime Minister, was officially mandated to make recommendations, there were communication gaps on the part of decision-makers. False information and rumors abounded, and without official denial, they tended to persist and intensify.

Intervention Pillar IV (“Effective Management of Mitigation Measures”) also failed, in part because of insufficient cross-sectoral collaboration in some committees. Most management bodies lacked administrative resources to ensure effective functioning and enforcement of procedures and timelines. These obstacles hindered the identification and implementation of effective global actions.

One of the authors of this paper was a member of the NCCMP and saw these difficulties firsthand. While the specific sociopolitical context in Burkina Faso may have contributed to what the author observed, it is also important to remember that, at that time, there was (and arguably remains) a scarcity of scientific evidence on the extent and consequences of the pandemic in sub-Saharan African societies. Our mission was to resolve tensions arising from divergent views between our roles as public health specialists, scientific researchers, and advisors to the government. The frames of reference for decision-making and the formulation of recommendations do not always follow the same logic and priorities, causing stress and dissonance within and between individuals.

Difficulties sometimes arose at multiple levels, and their entanglement hampered the effective implementation of certain measures. Vaccination against COVID-19 is one such example. While the NCCMP was committed to establishing the broad outlines of the strategy (priority target groups, vaccination sites, etc.), the international community demonstrated a shameless individualism that blocked supply chains. The disengagement of the “partners” was compounded by the challenges faced by the healthcare system in guaranteeing the safe delivery of vaccines outside the major cities. Finally, despite awareness-raising campaigns, vaccine resistance grew rapidly – misinformation campaigns represent a new barrier which remains largely understudied in the Burkinabe context.

### Facilitating Elements

The implementation of a response plan with such a large multisectoral dimension was seen as a first for the country, and the difficulties encountered throughout the recommendation and implementation processes constitute a learning experience for the stakeholders to guide future actions. In this sense, the spontaneous outpouring of solidarity at the national level to support the response was a major advantage.

Despite these efforts, implementation has often been burdened by limited financial resources; however, this did not prevent the mobilization of regional committees throughout the year, with the involvement of technical and financial partners at the local level. Another facilitating element was the commitment of a variety of individuals including artists, religious and community leaders, and civil society representatives to support government initiatives. This commitment was manifested in different ways including donations, sensitization, and promotion of the observance of preventive measures in places of worship. These elements promoted a spirit of solidarity and encourage the acceptability of preventive and protective measures.

### Recommendations to Improve the Strategy

Now that the COVID-19 pandemic has come to an end, some lessons to be learned could help us to better prepare for future health crises. Most importantly, the strengthening of the crisis response system must be considered from a dynamic, longitudinal, and collaborative perspective. The COVID-19 experience suggests that pandemic response plans must take into account the multisectoral nature of the evolving crisis and continue to incorporate new initiatives as the pandemic progresses.

Also, it is important to further harmonize national and regional mechanisms. While the NCCMP served as a catalyst for change in the national response model, regional committees remained within the traditional epidemic response model and failed to consider socioeconomic consequences in their interventions. To strengthen the response mechanism, links should be established between the sectoral committees and the regions; for example, regions could designate focal points for each sectoral committee.

Another lesson is that it is crucial that funds allocated to manage a particular pandemic be used to strengthen the health system on a sustainable basis, particularly in certain areas like emergency services, maintenance of high-value medical equipment, and triage services. While emergency measures provided Burkina Faso with much-needed therapeutic management equipment, a sustainable, long-term vision to optimize the health system was not articulated. Staff training, equipment maintenance, and a reliable supply of consumables must be integrated into pandemic response plans, and the work of the NCCMP should extend beyond the end of any crisis.

A national, public coordination unit for the management of health crises and disasters is essential. The existing system (the country’s emergency organization plan, known as the ORSEC plan) is piloted by the territorial administration and under the responsibility of the national fire department. The frequency, complexity, and severity of the epidemics in the region require a coordination unit with well-established mechanisms and resources to bring together multisectoral expertise and respond rapidly to health crises. Burkina Faso has experienced a relatively modest impact from the last two major disease outbreaks in West Africa, the 2014–2016 Ebola epidemic and the recent COVID pandemic,^33^ but the pressures of globalization, such as urbanization and climate change, affect the mobility of people, vectors, and pathogens and increase the likelihood of new acute health crises.^34^ The response to the COVID-19 pandemic illustrates the importance of having pre-existing operational channels to facilitate necessary interventions simultaneously and cohesively throughout multiple sectors.

## Particularities of Burkina Faso’s Response

While the public health measures against the COVID-19 pandemic have been examined in several sub-Saharan African countries (including West Africa), policy responses at the strategic level remain under-documented.^35–37^ The case of Burkina Faso stands out in two respects: on the one hand, in terms of the influence of community dynamics and, on the other, in terms of an economic recovery plan that took into consideration the significant socio-economic inequities and the risk of their deterioration.

Indeed, the initial COVID-19 response plan had not been drawn up in consultation with civil society partners, leading to community resistance to some public health measures. Although empirical evidence is lacking, it is plausible that this resistance was encouraged by the perception that COVID-19 was a disease of the elite, rich, white people or international travelers – it has been locally reported as the disease of those under air conditioning.^38^ This opposition contributed not only to reverse certain measures (and, arguably, to limit their harmful effects), but also to refocus on vulnerable strata of the population that had not been explicitly targeted by the government’s response plan. Significantly, in the face of such resistance in Burkina Faso, the strategic advisory committee modified its approach and became more inclusive. Religious and customary communities as well as local partners were increasingly involved in the planning and implementation of public health measures against the pandemic. It is therefore through its implementation, through practice, that the intervention plan has been adapted and made more inclusive. Earlier drafts, notably to prepare for a possible Ebola epidemic, had remained theoretical exercises that had never left the shelf.

This shift was also reflected in the countermeasures that were implemented to limit the detrimental effects of the restrictions that had been put in place by the committee itself. Two weeks after the adoption of decrees restricting people’s mobility and activities, the committee recommended a series of measures designed to mitigate the impact of these restrictions on the living conditions of the less well-off. These measures included: tax measures in favor of small and medium-sized businesses; securing stocks of staple products (sugar, milk, rice, oil, soap, etc.), with reinforced measures to combat clandestine storage and to control prices throughout the country; direct assistance to traders and market workers; and subsidies for access to two basic services, namely water and electricity. A substantial proportion of these measures focused on urban populations, despite reminders that rural areas “must not be forgotten”. Actions to support particularly vulnerable and rural populations were carried out jointly by the government, members of civil society and development partners. One example is the rapid return of door-to-door campaigns in communities, notably for seasonal malaria chemoprophylaxis and routine child immunization – a study recently showed that the COVID-19 did not contribute to reduce immunization coverage for infants.^39^

## Conclusion

Despite a seemingly lighter burden than that recorded by countries in Europe, Asia, or America, Burkina Faso has been impacted by COVID-19 through various mechanisms. From the onset of the crisis, it was evident that the health system was ill-equipped to detect and monitor an epidemic of respiratory infectious disease (despite the presence of a pilot surveillance system specifically designed for that purpose), let alone respond to it. Given the inability to deploy effective medical interventions on a national scale, a set of preventive measures was implemented to improve the health of the population; however, they represented an outdated, narrow, primarily biomedical vision of public health.^40^ The repercussions of these initial measures had negative, often unexpected consequences in other sectors, demonstrating the need for broader, decompartmentalized efforts.

For Burkina Faso, a national strategic multisectoral committee is working to lay the foundations of a new paradigm: a “new public health”^41^ that is no longer at the service of (or governed by) medicine but is resolutely interdisciplinary. The COVID-19 crisis has revealed that a wide range of societal aspects, including economic, environmental, educational, social, and psychological, are affected by measures taken to reduce the spread of disease, so approaches must be multisectoral to be truly beneficial to all individuals.

With only limited hindsight, lessons continue to emerge from the pandemic and how it was handled. In the space of 5 years, West Africa has experienced two major health crises. With the acceleration of globalization, it is counterproductive and unjust to approach this issue as one that does not extend beyond borders, and the race for vaccines by governments is the most recent example. The national interest of the major powers is to ensure optimal vaccine coverage within their borders; it was only once this was achieved that they considered improving the supply of vaccines to low-income countries. The scant number of vaccine doses distributed through the COVAX mechanism is, in this respect, indicative of the failure of multilateralism.^42^ This strategy exacerbates the unequal access of populations to vaccines and increases the risk of outbreaks of new and re-emerging diseases, including possible vaccine-resistant SARS-COV-2 variants, in under-vaccinated regions of the world.

While much attention is devoted in low-income countries to improving health systems, it is imperative to redirect efforts toward strengthening public health systems, as an extension of the State apparatus.^43^ Indeed, the pandemic illustrated that in order to manage future crises effectively and across sectors, States need permanent, public coordination units that act as an interface between sectors and have the authority, legitimacy, resources and established mechanisms to rapidly mobilize a variety of structures and actors at different levels of decision-making and implementation. These units should be able to work together quickly and efficiently, especially in the Sahelian sub-region. At a time when global health governance has been sorely deficient, there is an opportunity to strengthen the mechanisms for collaboration between a core group of Sahelian countries – even if it means gradually capitalizing and expanding to the entire region of sub-Saharan Africa. More than 400°years ago, plague epidemics contributed to the emergence and institutionalization of the State in Europe.^44–46^ The COVID-19 pandemic should serve as a reminder of the importance of ensuring equal capacity and consideration of multiple levels of government in coordinating in their responses to public health crises.
